# Documenting and explaining the HIV decline in east Zimbabwe: the Manicaland General Population Cohort

**DOI:** 10.1136/bmjopen-2017-015898

**Published:** 2017-10-06

**Authors:** Simon Gregson, Owen Mugurungi, Jeffrey Eaton, Albert Takaruza, Rebecca Rhead, Rufurwokuda Maswera, Junior Mutsvangwa, Justin Mayini, Morten Skovdal, Robin Schaefer, Timothy Hallett, Lorraine Sherr, Shungu Munyati, Peter Mason, Catherine Campbell, Geoffrey P Garnett, Constance Anesu Nyamukapa

**Affiliations:** 1 Department of Infectious Disease Epidemiology, Imperial College London, London, UK; 2 Biomedical Research and Training Institute, Harare, Zimbabwe; 3 Zimbabwe Ministry of Health and Child Care, Harare, Zimbabwe; 4 University of Copenhagen, Copenhagen, Denmark; 5 University College London, London, UK; 6 London School of Economic and Political Science, London, UK

**Keywords:** HIV & AIDS, HIV decline, Demographic impact, HIV incidence, Zimbabwe, Sexual behaviour change

## Abstract

**Purpose:**

The Manicaland cohort was established to provide robust scientific data on HIV prevalence and incidence, patterns of sexual risk behaviour and the demographic impact of HIV in a sub-Saharan African population subject to a generalised HIV epidemic. The aims were later broadened to include provision of data on the coverage and effectiveness of national HIV control programmes including antiretroviral therapy (ART).

**Participants:**

General population open cohort located in 12 sites in Manicaland, east Zimbabwe, representing 4 major socioeconomic strata (small towns, agricultural estates, roadside settlements and subsistence farming areas). 9,109 of 11,453 (79.5%) eligible adults (men 17-54 years; women 15–44 years) were recruited in a phased household census between July 1998 and January 2000. Five rounds of follow-up of the prospective household census and the open cohort were conducted at 2-year or 3-year intervals between July 2001 and November 2013. Follow-up rates among surviving residents ranged between 77.0% (over 3 years) and 96.4% (2 years).

**Findings to date:**

HIV prevalence was 25.1% at baseline and had a substantial demographic impact with 10-fold higher mortality in HIV-infected adults than in uninfected adults and a reduction in the growth rate in the worst affected areas (towns) from 2.9% to 1.0%pa. HIV infection rates have been highest in young adults with earlier commencement of sexual activity and in those with older sexual partners and larger numbers of lifetime partners. HIV prevalence has since fallen to 15.8% and HIV incidence has also declined from 2.1% (1998-2003) to 0.63% (2009-2013) largely due to reduced sexual risk behaviour. HIV-associated mortality fell substantially after 2009 with increased availability of ART.

**Future plans:**

We plan to extend the cohort to measure the effects on the epidemic of current and future HIV prevention and treatment programmes. Proposals for access to these data and for collaboration are welcome.

**Strengths and limitations of this study**

- The Manicaland cohort is one of a handful of long-running, large-scale general population HIV sero-surveys conducted in countries in sub-Saharan Africa with widespread epidemics that constitute a key resource for evaluating the population-level impact of HIV prevention and treatment programmes.
- The current data span the period 1998 to 2013 during which Zimbabwe experienced one of the largest HIV epidemics in the world and was almost unique in sub-Saharan Africa in achieving a substantial national decline in HIV prevalence largely caused by reductions in sexual risk behaviour. The study data also cover periods prior to, during and following the roll-out of prevention of mother-to-child transmission services (introduced in Zimbabwe from 2002) and antiretroviral treatment services (from 2004 with rapid scale-up from 2009).
- The study data include comprehensive and consistent measurements of trends in HIV prevalence, HIV incidence, HIV-associated and all-cause mortality, sexual risk behaviours, health-seeking behaviours, and in the coverage and effects of national HIV control programmes over time. The study also includes parallel measurement of trends in HIV prevalence among pregnant women attending local antenatal clinics which permits assessment of biases in the primary source of routine HIV surveillance data used by countries and Joint United Nations Programme on HIV/AIDS to produce national and regional HIV estimates.
- Findings from the study are generalisable to Zimbabwe as a whole and data are available on their wider generalisability.
- Limitations of the cohort include the age limit (55 years) for participation, changes in eligibility criteria across rounds, and long intervals (2–3 years) between rounds of follow-up such that short-term migrants may be missed and measurement of some key variables including mortality can be subject to recall and misclassification bias.

## Introduction

The Manicaland general population open cohort HIV sero-survey (Manicaland cohort) was set up in 1998 by researchers from Imperial College London and the Biomedical Research and Training Institute (BRTI) with funding from the Wellcome Trust. Findings from an earlier study (1993–1996) had shown that HIV was spreading extensively in rural areas of eastern Zimbabwe, and was associated with large increases in mortality.^1^ The new cohort was established to provide robust scientific data on HIV prevalence and incidence within a general population sample, on local patterns of sexual behaviour and their role in the spread of HIV, and on the mortality and wider demographic impact of HIV in a range of different settings in Manicaland, Zimbabwe’s eastern province.

Following an initial pilot study,^2^ in the first two rounds of the cohort study, a two-arm cluster-randomised controlled trial was conducted of a peer education, condom distribution and syndromic management of sexually transmitted infections (STIs) intervention in female sex workers and male clients to reduce the spread of HIV infection. The trial found that this intervention was not effective in reducing HIV incidence within the general population.^3^

In subsequent rounds of the cohort survey, the research aims were extended to include investigation of the temporal dynamics of the HIV epidemic, the social determinants of HIV, and the coverage and effectiveness of national HIV control programmes, including antiretroviral therapy (ART) services introduced in the mid-2000s.

## Cohort description

### Study design and location

**Figure 1:**
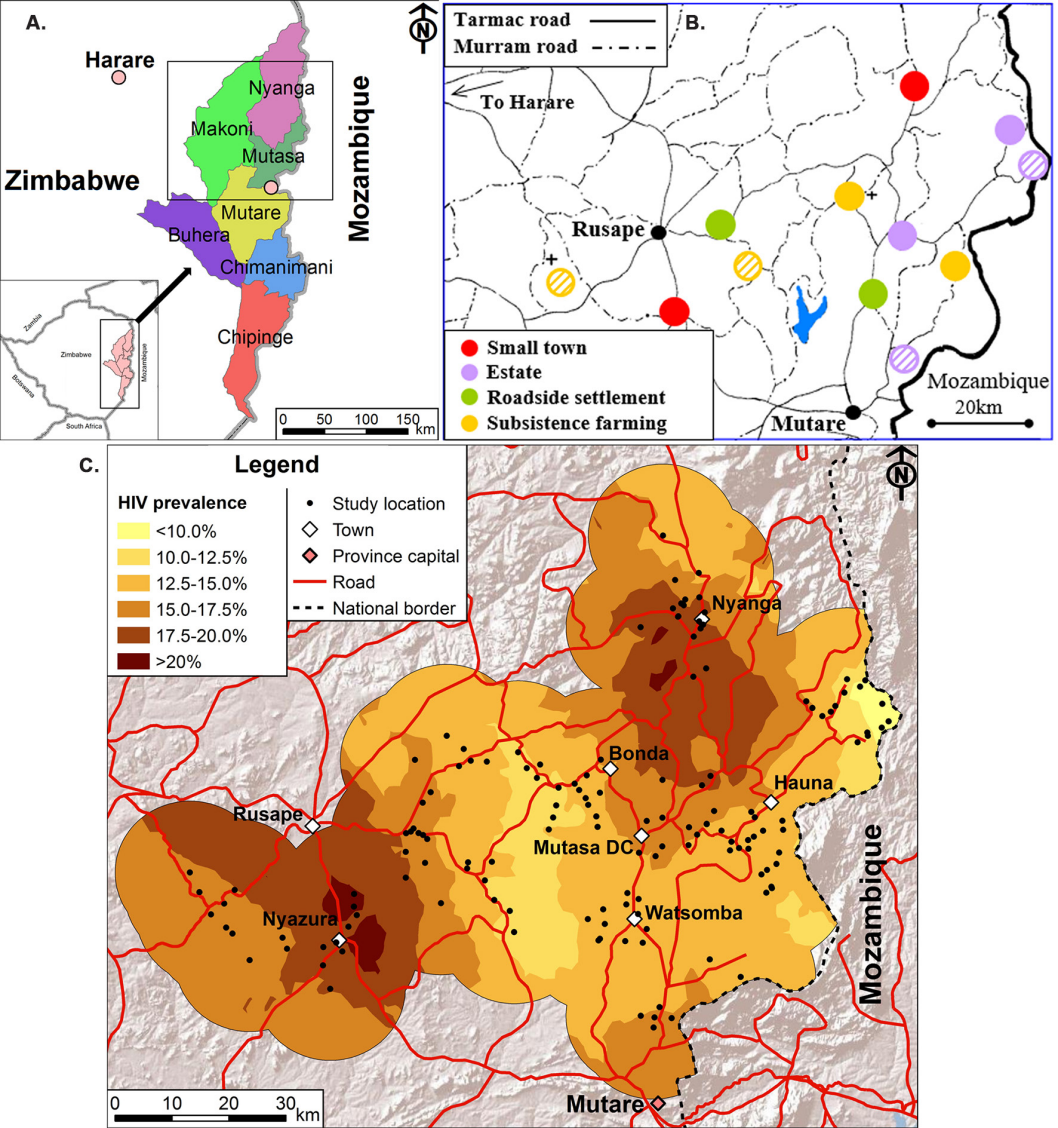
(A) Location of the study districts in Manicaland province, east Zimbabwe; (B) map showing the 12 study areas in Manicaland province with the four sites excluded from round 6 shown with shading; (C) map of HIV prevalence across the study areas showing the study villages, estate compounds and urban locations at round 5 (2009–2011).

The study is designed as a stratified General Population Open Cohort HIV Sero-Survey and is located in three districts (Mutasa, Makoni and Nyanga) of Manicaland province, which runs along Zimbabwe’s eastern border with Mozambique (figure 1A).

To accommodate the two-arm cluster-randomised trial, a stratified design was chosen with six pairs of sites (figure 1B) matched on socioeconomic criteria. Consequently, the Manicaland cohort was drawn from two small towns (Nyanga and Nyazura), four agricultural estates (Katiyo and Eastern Highlands tea estates and Selborne and Sheba forestry plantations), two roadside settlements (Watsomba and Nyabadza/Nyahukwe), and four subsistence farming areas (Bonda, Honde, St. Theresa’s and St. Killian’s missions).

The central coordinates of the component study locations (rural village markets, estate compounds and urban locations) have been mapped using handheld global positioning system devices (figure 1C). Overall, the study sites are located between latitudes −18.07^o^ and −18.85^o^ and longitudes 31.93^o^ and 33.04^o^, an average distance of 180.8 km (range: 126.1–219.6 km) and 58.9 km (13.3–99.3 km) from Harare and Mutare, the national and provincial capitals, and encompass a combined area of 8184 km^2^.

The study areas are located in the Eastern Highlands region of Zimbabwe (average altitude approximately 1300 m) and are predominantly rural but benefit from a temperate climate (quite hot with rains between October and March; cool and dry from May to August) with generally relatively good rainfall (average temperature and annual rainfall c25° and c1000 mm) and fertile soils. The principal crops include maize, sorghum, finger millet, yams, cotton, tea, bananas, avocados, sugarcane and other fruits. Most local people also grow vegetables and keep cattle, goats and chicken.

### Eligibility criteria and participation and follow-up rates

**Figure 2:**
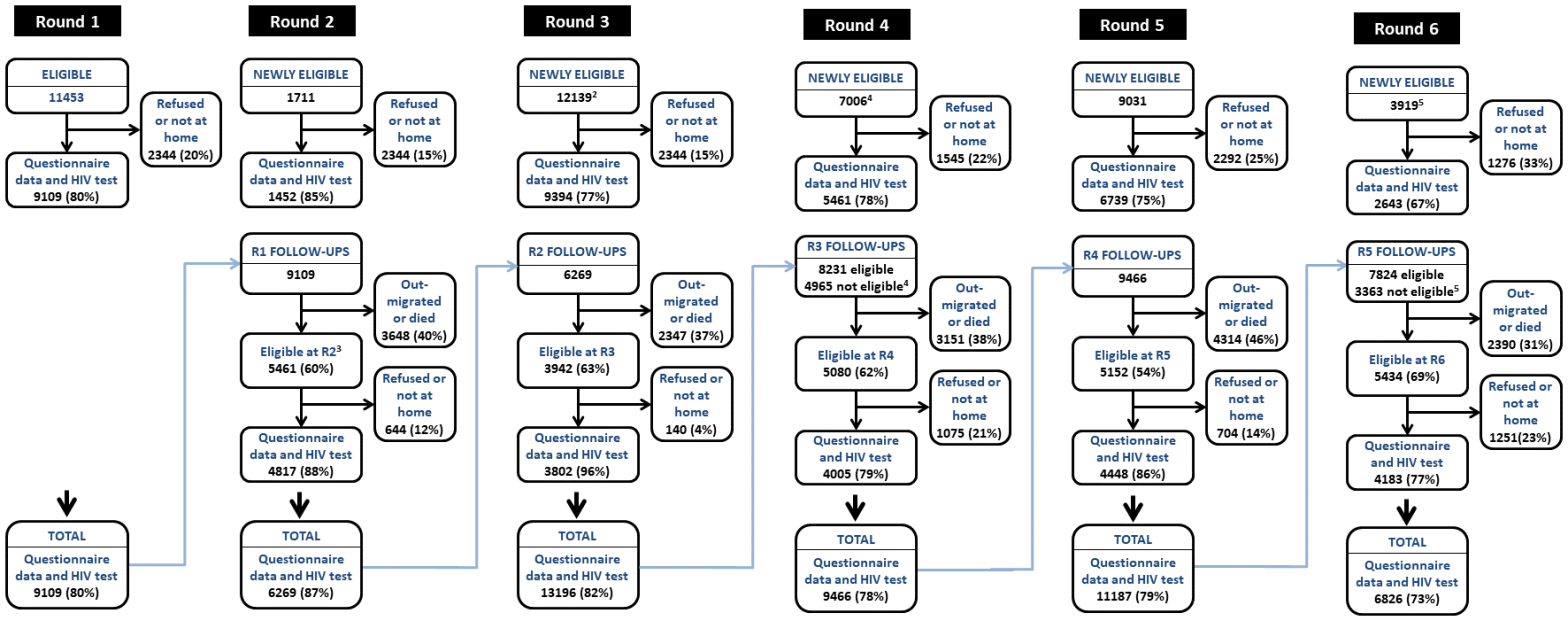
Flow diagram showing individual participation rates and follow-up rates in the cohort by survey round. Notes: (1) Participation and follow-up rates based on eligibility criteria at round 1: men aged 17–54, women aged 15–44; regular members of households in the study areas; stayed in the household at least 4 nights in the last month. (2) In rounds 1 and 2, a maximum of one member per marital grouping was selected at random for interview; this restriction was dropped from round 3 onwards. (3) In round 2, individuals who migrated into a household since round 1 were only treated as eligible from site 5 (out of 12 sites). (4) From round 4, eligibility for individual interviews was restricted to individuals from a random sample of two-thirds of enumerated households. (5) In round 6, the number of study sites was reduced from 12 to 8 (2 agricultural estates and 2 subsistence farming areas were dropped). R1, R2, … indicate round numbers in the cohort survey.

The baseline census and survey were conducted in a phased manner (one site at a time) between July 1998 and January 2000. In the census, a household was defined as a group of people who regularly eat together from the same cooking pot. Regular household members aged 17–54 years for men and 15–44 years for women—the ages of expected highest HIV incidence—were eligible for enrolment into the cohort (figure 2). However, participation in the cohort was restricted to a maximum of one member of each marital group (ie, a man and his wife or wives), selected at random, in order to maximise statistical power for the trial of the peer education and STI treatment intervention. Local village community workers were employed as guides to assist in locating participants. Where eligible individuals were unavailable for interview at the first household visit, appointments and up to two additional visits were made.

The second round of the open cohort survey was conducted between August 2001 and July 2003. All baseline respondents and individuals who had aged into the qualifying age range were eligible for this round. In-migrants in the 3-year intersurvey period and visitors were eligible for enrolment in the last eight sites. The third round ran from August 2003 to August 2005. Eligibility for the cohort was extended to include all men and women aged 15–54 years and the restriction to one member of each marital group was lifted. The fourth round ran from August 2006 to November 2008. All households were eligible for enumeration in the census and the same age criteria were used but follow-up and recruitment into the cohort were limited to members of a random sample of two-thirds of households due to funding constraints. The same eligibility criteria were applied in round 5, which ran from October 2009 to July 2011, and in round 6, which ran from July 2012 to November 2013. In round 6, four sites were dropped from the study, again due to funding constraints.

In the household censuses, the overall response rate was 98.2% (8233/8386) at baseline (1998–2000), 97.1% (6982/7189) in round 2 (2001–2003), 95.4% (9322/9773) in round 3, 93.7% (11865/12668) in round 4, 98.0% (13180/13453) in round 5 and 90.9% (8116/8931) in round 6 (2012–2013) (see online supplementary table S1). This estimate for the response rate in the baseline census is an overestimate because fears that the researchers were Satanists—because they were asking for blood specimens thought to be used by Satanists—caused difficulties in identifying households. From the third round onwards, a steady increase in numbers of households has been observed reflecting reductions in these Satanist fears and growth in the population. The escalating economic crisis and a government initiative in 2005 to clean up the urban areas contributed to the increase in household numbers in round 4 (2006–2008). No difference in response rates was seen between households selected and not selected for individual interviews in rounds 4 to 6 (see online  supplementary table S1).

10.1136/bmjopen-2017-015898.supp1 Supplementary material 1

At baseline, 11 453 individuals were eligible for the study, of whom 79.5% (9109) participated (figure 2, see online supplementary figure S2). In subsequent rounds, using the same age ranges for each sex for comparison (17–54 years for men; 15–44 years for women), overall participation rates have been similar except in round 6 when the overall rate fell to 73.0%. The cohort size has varied between 6269 in round 2 (2001–2003) and 13 196 in round 3 (2003–2005) reflecting, primarily, the changes in eligibility criteria between rounds. Participation rates generally have been higher in women than in men (see online supplementary table S2). Direct refusal rates are consistently low (<5%), most non-participation being due to temporary absences from the household reflecting the high population mobility found in Zimbabwe.

The follow-up rate among all members of the cohort in the preceding round has varied between rounds from 47.0% (2006–2008 to 2009–2011) and 60.6% (2001–2003 to 2003–2005) (figure 2, see online supplementary table S1). However, most loss to follow-up comprises previous members of the cohort who ceased to be eligible due to death or out-migration from the study areas. Among those who remained eligible, cohort follow-up rates have been high, ranging from 77.0% (2009–2011 to 2012–2013) to 96.4% (2001–2003 to 2003–2005). Follow-up rates have differed little between HIV-positive and HIV-negative individuals.

### Questionnaire data

In the household census questionnaire, the location and identity of each household is recorded (table 1). Basic sociodemographic information is collected for each member of the household including information on the eligibility criteria for inclusion in the cohort for adults. In households enumerated in previous rounds of the census, details of individuals who stayed in the household at or following the last visit are recorded (even if they have since left the household) and details of their survival status and date of leaving the household (where applicable) are recorded. Information on moveable and immoveable household assets is also collected for use in measuring socioeconomic status using wealth indices.^4^

**Table 1:** Information collected in the household census, individual interviews, verbal autopsy interviews in the Manicaland Cohort Survey

Survey instrument Variable(s)	Survey rounds*	Scope of question	Specific information
Household census			
Household ID	All	Each household	District, village name, household head
Household status	All	Each household	New or dissolved
Pre-existing household members	All	Each household	Name, relationship to household head, sex, age, education, parents’ survival status, member’s survival status, nights spent in household in the last month, whether selected for interview
New household members	All	Each household	As above plus date joined household
Former household members	All	Each household	Survival status, date and reason for leaving household, current residence (for out-migrants)
Household assets	All	Each household	Water source, toilet type, house type, moveable assets
GPS coordinates	Round 6	Collected at village level only	GPS coordinates for central market area
Individual interviews			
Background characteristics	All	Random sample of adult household members†	Sex, age, parents' survival (<30 years), education, migration, religion, male circumcision, employment, substance use, marital history and status, participation in community groups
Spouse characteristics	All	Up to four spouses	Age, age at marriage, cohabitation, education, employment, HIV test and disclosure, migration, religion, male circumcision
Psychological health	Rounds 5 to 6	All selected adults	Variables for Shona Symptom Questionnaire and WHO Questionnaire
Sexual relationships	All	All selected adults	Age at first sex, regular/non-regular partners, condom use, partner loops, concurrency, commercial/transactional sex, informal confidential voting interviews used for literate participants
Health and access to treatment	Rounds 3 to 6	All selected adults	General health, healthcare behaviour, STDs, HIV testing, disclosure, CD4 counts, ART initiation / adherence, side effects, palliative care
HIV awareness and impact	All	All selected adults	Knowledge, risk perception, self efficacy, stigma, masculinity, exposure to HIV prevention
Fertility history	All	All women	Sex, date of birth, PMTCT uptake, survival status, date of death
Pregnancy history	All	Current and recent pregnancies (last 3 years)	Antenatal care, PMTCT uptake, infant diagnosis, breastfeeding, postpartum amenorrhoea, sexual abstinence, family planning
HIV infection status	All	All selected adults	Combaids HIV-1/HIV-2 dipstick test; potential seroconversions, confirmed with Vironostika HIV Uniform-II plus O
Verbal autopsy interviews			
Social circumstances	Round 2→	Deaths in the cohort	Relationship of caregiver/respondent to deceased, sex, age, date of death, HIV testing and ARV treatment /adherence, history of deceased; deceased’s spouse’s status, household impact
Financial implications		Deaths in the cohort	Healthcare costs and funding contributions, impact of illness, on employment, pension/termination payments, widow’s pension
Effects on deceased’s children		Births before and since deceased’s last interview	Survival status, PMTCT, orphanhood, education, care arrangements
Medical conditions and accidents		Deaths in the cohort	Accidents, homicide, suicide
Maternal mortality		Female deaths	Symptoms of maternal mortality
Final illness		Deaths in the cohort	Symptoms of final illness

*Dates for rounds of the household census and individual cohort: round 1: July 1998 to January 2000; round 2: August 2001 to July 2003; round 3: August 2003 to August 2005; round 4: August 2006 to November 2008; round 5: October 2009 to July 2011; round 6: July 2012 to November 2013.

† Eligibility for the adult individual general population cohort: round 1: regular household members who had slept in the household at least four nights in the last month and had been resident in the household at the same time 1 year earlier, men aged 17–54 years and women aged 15–44 years limited to one member of a marital union selected at random (to maximise power in the embedded community randomised controlled trial of HIV prevention interventions); round 2: same criteria as in round 1 except that in-migrants were not eligible in the first four sites (Katiyo tea estate, Eastern Highlands tea estates, Bonda Mission, Honde Mission). In the remaining eight sites (and in all sites in subsequent rounds), individuals who stayed in households in the study areas the night before the census visit but who had not met the round 1 residence tests were treated as eligible for participation in the cohort; round 3: eligible age ranges extended to 15–54 years for men and women; restriction to one member of each marital union dropped and residence criteria extended to all persons who slept in the household the previous night rounds 4 and 5: same criteria as in round 3 except that eligibility was limited to adults in a random sample of two-thirds of households in the household census; round 6: same criteria as in rounds 4 and 5 but restricted to eight sites: Eastern Highlands tea estate, Bonda Mission, Honde Mission, Selborne forestry estate, Nyazura, Nyanga, Watsomba, Nyabadza/Nyahukwe.

ART, antiretroviral therapy; PMTCT, prevention of mother-to-child transmission of HIV infection; STDs, sexually transmitted diseases.

The individual respondent questionnaires used for the Manicaland cohort comprise sections on the respondent’s own sociodemographic characteristics, the characteristics of up to four current spouses, the respondent’s psychological health (since round 5—including all questions from the Shona Symptom Questionnaire,^5^ a locally validated common mental health inventory and WHO’s Self-Report Questionnaire^6^), sexual relationships, health and access to treatment (since round 3), HIV knowledge and awareness (including exposure to HIV control programmes), and fertility and pregnancy histories (table 1). Dried blood spot (DBS) specimens have been collected for anonymised HIV testing for research purposes only as a requirement for participation at each round of the cohort. Free parallel voluntary HIV counselling and testing services were made available locally for cohort members during survey visits.^7^

For cohort members who passed away between rounds of the survey, verbal autopsy interviews were conducted with the deceased’s primary caregiver. The questionnaire included questions on accidents, medical conditions and symptoms during the final illness,^8^ and on social and financial circumstances surrounding the death^9^ (table 1).

Unusual features of the Manicaland cohort include parallel HIV surveys among pregnant women attending antenatal (ANC) check-ups at local health clinics, conducted to obtain information in biases in routine HIV surveillance data^10^ (see online supplementary table S4); and six rounds of facility surveys conducted (2010–2016) to measure trends in local availability of HIV services.^11^

In the first five rounds of the Manicaland cohort, all interviews were conducted using paper questionnaires. In round 6, the questionnaires for household census and individual cohort interviews were administered using HTC Smartphones using EpiCollect software.^12^ Copies of the study questionnaires are available from the Manicaland Centre for Public Health Research website (http://www.manicalandhivproject.org/questionnaires). The data from all rounds of the study are held in an SQL relational database developed for use in Microsoft Access.

### Data on HIV infection rates

New participants in the cohort at each round provided DBS specimens that were tested for HIV infection at the BRTI laboratory in Harare, using a consistent testing strategy^13^ across all rounds of the survey. At each round of follow-up, HIV-negative individuals from the previous round were retested for HIV infection using newly collected DBS specimens and the same testing strategy. The HIV testing strategy used a dipstick dot-EIA test as the screening test (the ICL dipstick dot-EIA (ICL-HIV 1&2 Dipstick, Thailand)) in round 1 and the Combaids dot-EIA (Combaids-HIV-1&2 Dipstick, Span Diagnostics, India) in rounds 2 to 6, and a third generation plate EIA (Abbott third generation HIV 1&2 EIA (Abbott Laboratories, USA)) or Genelavia MIXT HIV1&2 (Sanofi Diagnostics Pasteur SA, France) in rounds 1 and 2; Vironostika HIV Uniform II in rounds 3 to 6) as the confirmatory test. Where the test results from successive survey rounds indicated a seroconversion, the sample from the first of these rounds was retested to confirm the original negative result using the same dipstick dot-EIA test. Where this result remained negative, the plate EIA test was run on the DBS specimens from both rounds to confirm the results. BRTI laboratory test results were routinely evaluated in the Zimbabwe National Quality Assurance Programme.

The HIV incidence rates for each intersurvey period reported in this paper were estimated assuming that new infections between rounds occurred midway between the first and second interview dates.

### Characteristics of the study population

The study population is comprised primarily of people who speak the *Manyika* dialect of Zimbabwe’s majority *Shona* language. Most are also Christian, belonging to a large number of different Mission, Apostolic, Pentecostal and other spiritual churches.^14^ Customary marriage, based on payment of bride-wealth, is almost universal, and is often followed by a church wedding. Polygyny remains common in some Apostolic churches and people who practice traditional religion.^15^ As elsewhere in Zimbabwe, education levels are high compared with other countries in sub-Saharan Africa.^16 17^

Just over half of the cohort is female reflecting the predominantly rural study areas (table 2). Over time, the cohort has aged somewhat (from a median of 25 years in round 1 to 27 years in round 5) and the proportion living on agricultural estates has fallen due to retrenchments on these estates reflecting increased mechanisation and the economic decline. The latter is also reflected in the large increase in unemployment between round 2 (32.8%) and round 4 (55.5%). However, education levels^17^ and the proportion of the cohort who are married have both increased due, in part, to the ageing of the cohort.

**Table 2:** Sociodemographic characteristics of cohort participants by survey round, Manicaland cohort, Zimbabwe, 1998–2013

Number of participants	1998–2000	2001-2003*	2003–2005	2006–2008	2009–2011	2012-2013†
	N	N	N	N	N	N
	9109	6269	13 196	9466	11 187	6826
Sex						
Male	4164 (45.7%)	2730 (43.6%)	5314 (40.3%)	3919 (41.4%)	4474 (40.0%)	2772 (40.6%)
Female	4945 (54.3%)	3539 (56.4%)	7882 (59.7%)	5547 (58.6%)	6713 (60.0%)	4054 (59.4%)
Age (years)						
15–24 (17–24 for men)	4300 (47.2%)	2765 (44.1%)	6039 (45.8%)	4075 (43.0%)	4394 (39.3%)	2444 (35.8%)
25–34	2630 (28.9%)	1820 (29.0%)	3973 (30.1%)	3072 (32.5%)	3633 (32.5%)	2263 (33.2%)
35–44	1832 (20.1%)	1408 (22.5%)	2639 (20.0%)	1912 (20.2%)	2622 (23.4%)	1741 (25.5%)
45–54 (men only)	347 (3.8%)	276 (4.4%)	545 (4.1%)	406 (4.3%)	538 (4.8%)	378 (5.5%)
Residence						
Small towns	1539 (16.9%)	978 (15.6%)	2174 (16.5%)	1578 (16.7%)	2010 (18.0%)	1759 (25.8%)
Agricultural estates	3005 (33.0%)	2095 (33.4%)	4022 (30.5%)	2663 (28.1%)	2992 (26.7%)	1418 (20.8%)
Roadside settlements	1530 (16.8%)	1090 (17.4%)	2493 (18.9%)	1789 (18.9%)	2246 (20.1%)	1917 (28.1%)
Subsistence farming villages	3035 (33.3%)	2106 (33.6%)	4507 (34.1%)	3436 (36.3%)	3939 (35.2%)	1732 (25.3%)
Migrant status						
In-migrant (<3 years)	2182 (23.9%)	788 (12.6%)	2282 (17.4%)	1716 (18.1%)	1299 (11.6%)	738 (10.8%)
Non-migrant	6927 (76.1%)	5481 (87.4%)	10 904 (82.6%)	7750 (81.9%)	9888 (88.4%)	6088 (89.2%)
School education						
None	271 (3.0%)	86 (1.4%)	9 (0.1%)	0 (0.0%)	2 (0.0%)	48 (0.7%)
Primary	3276 (36.0%)	1970 (31.4%)	3692 (28%)	2150 (22.7%)	2272 (20.3%)	1314 (19.2%)
Secondary	5394 (59.2%)	4097 (65.4%)	8954 (67.8%)	6968 (73.6%)	8560 (76.5%)	5265 (77.1%)
Higher	164 (1.8%)	53 (0.8%)	198 (1.5%)	180 (1.9%)	245 (2.2%)	154 (2.3%)
Missing	4 (0.0%)	63 (1.0%)	343 (2.6%)	168 (1.8%)	108 (1.0%)	45 (0.7%)
Marital status						
Single	3391 (37.2%)	2274 (36.3%)	4431 (33.6%)	3128 (33.0%)	3149 (28.2%)	1782 (26.1%)
Married	4537 (49.8%)	3280 (52.3%)	7110 (53.9%)	5138 (54.3%)	6776 (60.6%)	4277 (62.6%)
Divorced or separated	762 (8.4%)	393 (6.3%)	996 (7.5%)	621 (6.6%)	640 (5.7%)	470 (6.9%)
Widowed	405 (4.5%)	309 (4.9%)	636 (4.8%)	456 (4.8%)	546 (4.9%)	279 (4.1%)
Missing	14 (0.1%)	13 (0.2%)	23 (0.2%)	123 (1.3%)	76 (0.7%)	18 (0.3%)
Employment status						
Formal sector	2344 (25.7%)	1725 (27.5%)	3037 (23.0%)	1982 (20.9%)	1942 (17.4%)	1246 (18.2%)
Informal sector	2920 (32.1%)	1493 (23.8%)	2568 (19.4%)	1015 (10.7%)	1620 (14.5%)	795 (11.7%)
Unemployed	3076 (33.8%)	2059 (32.8%)	5870 (44.5%)	5252 (55.5%)	6225 (55.6%)	3786 (55.5%)
Student	755 (8.3%)	992 (15.8%)	1698 (12.9%)	1194 (12.6%)	1394 (12.5%)	824 (12.1%)
Missing	14 (0.1%)	0 (0.0%)	23 (0.2%)	23 (0.2%)	6 (0.0%)	175 (2.6%)
HIV-positive						
12 original sites	2127 (23.4%)	1337 (21.3%)	2533 (19.2%)	1657 (17.5%)	1795 (16.1%)	-
8 sites in round 6	1502 (25.1%)	964 (23.3%)	1784 (19.9%)	1230 (18.0%)	1306 (16.7%)	1065 (15.8%)‡

To provide a consistent comparison across rounds of the cohort survey, these data are shown throughout for men aged 17–54 years and women aged 15–44 years who were regular household members and stayed in the household for at least 4 nights in the last month before the interview.

*In round 2, individuals who had migrated into a household in the study areas since baseline were only treated as eligible from site 5 (out of 12 sites).

†In round 6, the number of study sites was reduced from 12 to 8 (2 agricultural estates and 2 subsistence farming areas were dropped).

‡Sixty missing cases due to indeterminate HIV test results.

## Findings to date

### HIV surveillance in a high HIV prevalence setting

**Figure 3:**
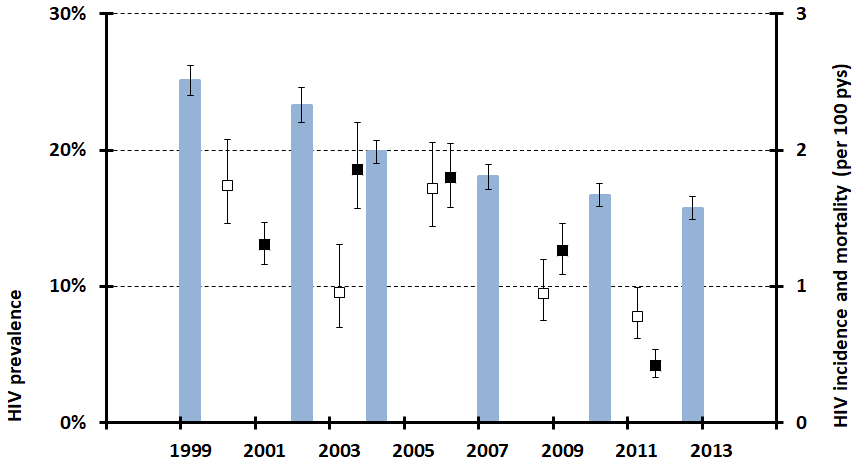
Trends in HIV prevalence (histogram), HIV incidence (open squares) and all-cause mortality (solid squares) in men aged 17–54 years and women aged 15–44 years resident in the eight sites included in all six rounds of the Manicaland general population open cohort sero-survey, Manicaland, Zimbabwe, 1998–2013. Whiskers indicate 95% CI. Pyrs indicates person years.

The pilot study provided important early evidence that, by the early 1990s, HIV prevalence had reached high levels (23.3%) in the general population in rural areas of Zimbabwe.^2^ Up to this point, HIV prevalence had been found to be much higher in urban areas than in rural areas in most sub-Saharan African countries and the high prevalence in rural areas meant that Zimbabwe was faced with one of the world’s largest HIV epidemics. The study also found extremely high prevalence in young women aged 15–24 years (20.8%).^2^ In the Manicaland cohort, HIV prevalence was 25.1% at baseline (1998–2000) and fell steadily to 16.7% in round 5 and 15.8% in round 6 (table 2, figure 3).

In the late 1980s, Zimbabwe established a national HIV surveillance system based on unlinked anonymous testing of pregnant women attending ANC check-ups.^18 19^ The Manicaland Study, using data from its parallel general population cohort and ANC HIV prevalence surveys, has contributed information on the extent and causes of bias in ANC surveillance data on levels and trends in HIV prevalence^10 20–22^ which have been used to develop the methods used in Zimbabwe and internationally to produce national HIV estimates.^23–26^

### Sexual behaviour, migration and the spread of HIV infection

Understanding the role of sexual behaviour in the spread of HIV infection has been hampered by reporting biases in the data.^27^ In the Manicaland cohort, we developed an informal confidential voting interview method to reduce social desirability bias which has produced epidemiologically plausible results.^28^ Data from the cohort were used to provide a detailed description of patterns of sexual risk behaviour in eastern Zimbabwe and their associations with HIV infection^13 29 30^ and of changes in behaviour over time (table 3). In particular, the data showed that large age differences between sexual partners were common in the study population (median difference 6 years for women aged 15–24 years; IQR 4–9 years) and were associated with increased risk of HIV infection in young people.^29^ Using a mathematical model, we found that age differences between men and women in sexual partnerships are unlikely to affect the scale of HIV epidemics but can explain the large female-male ratios of HIV infection found in young adults in sub-Saharan African populations.^31^ Data from the cohort provided evidence that medical injections are not a major contributor to new HIV infections in generalised epidemics.^32^

**Table 3:** Trends in sexual behaviour reported by men and women in the Manicaland cohort, 1998 to 2013^a^

Survey period	Age at first sex*		Multiple sexual partners†		Casual sexual partner(s)†		Condom use with casual partners‡		New partner in the last 12 months		Commercial sex§		Concurrent partners¶	
	Median (IQR)	N	% (95% CI)	N	% (95% CI)	N	% (95% CI)	N	% (95% CI)	N	% (95% CI)	N	% (95% CI)	N
Men														
1998–2000	18.6 (16.9 to 20.5)	1319	–	–	–	–	–	–	49.3 (47.3 to51.4)	2322	18.6 (17.0 to20.2)	2341	17.6 (16.0 to19.2)	2323
2001–2003	19.1 (17.5 to 21.4)	771	50.5 (47.8 to53.1)	1443	48.5 (45.9 to51.1)	1443	21.2 (18.3 to24.5)	692	33.2 (30.8 to35.7)	1446	9.0 (7.6 to10.6)	1445	10.5 (9.0 to12.2)	1444
2003–2005	19.4 (17.7 to 21.6)	1590	42.2 (40.4 to44.0)	2946	46.1 (44.3 to48.0)	2946	26.4 (24.1 to28.9)	1331	34.7 (33.0 to36.4)	2944	8.2 (7.3 to9.3)	2934	9.1 (8.1 to10.2)	2946
2006–2008	20.2 (18.2 to 22.4)	1188	41.5 (39.5 to43.6)	2227	40.3 (38.3 to42.4)	2231	28.1 (25.1 to31.3)	835	28.6 (26.7 to30.5)	2230	6.4 (5.4 to7.4)	2264	7.4 (6.4 to8.6)	2247
2009–2011	22.0 (19.6 to 24.4)	1085	32.4 (30.5 to34.3)	2313	28.3 (26.4 to30.1)	2315	32.9 (29.2 to36.9)	598	20.2 (18.6 to21.9)	2313	2.4 (1.8 to3.1)	2315	7.4 (6.4 to8.5)	2315
2012–2013	21.0 (19.0 to 23.2)	920	41.1 (38.9 to43.2)	2014	38.1 (36.0 to40.2)	2014	35.4 (31.7 to39.1)	676	27.7 (25.8 to29.7)	2011	2.9 (2.2 to3.7)	2069	8.7 (7.5 to10.0)	2027
Women														
1998–2000	18.8 (17.3 to 20.5)	1421	–	–	–	–	–	–	20.8 (19.2 to22.4)	2614	5.0 (4.2 to5.9)	2644	2.8 (2.2 to3.5)	2610
2001–2003	19.0 (17.7 to 20.5)	992	12.6 (11.1 to14.2)	1759	11.4 (10.0 to13.0)	1760	10.0 (6.2 to15.0)	200	13.5 (12.0 to15.2)	1760	3.2 (2.5 to4.2)	1759	1.3 (0.8 to2.0)	1759
2003–2005	19.0 (17.6 to 20.7)	2450	9.2 (8.4 to10.2)	4165	11.6 (10.7 to12.6)	4165	11.7 (8.8 to15.1)	428	12.2 (11.2 to13.3)	4165	2.6 (2.1 to3.1)	4165	0.7 (0.5 to1.0)	4165
2006–2008	19.4 (17.7 to 21.0)	1719	15.4 (14.2 to16.8)	3044	16.2 (14.9 to17.6)	3047	10.9 (8.0 to14.4)	386	18.8 (17.4 to20.2)	3055	4.9 (4.1 to5.7)	3096	0.8 (0.5 to1.2)	3076
2009–2011	19.1 (17.7 to 20.8)	1926	7.5 (6.7 to8.4)	3785	8.1 (7.3 to9.0)	3786	9.6 (6.2 to13.9)	250	9.0 (8.1 to10.0)	3788	2.4 (2.0 to3.0)	3792	0.7 (0.4 to1.0)	3787
2012–2013	18.8 (17.3 to 20.5)	1436	7.9 (6.9 to8.8)	3183	6.7 (5.8 to7.6)	3184	34.4 (24.9 to45.0)	93	10.1 (9.1 to11.2)	3186	3.2 (2.7 to3.9)	3240	1.0 (0.7 to1.4)	3190

*Life-table survival estimates based on reports from respondents aged under 25 years.

†More than one sexual partner or at least one casual partner in the last 3 years (last 2 years for 2003–2005).

‡Consistent condom use with all casual partners in the last 3 years.

§Based on responses to a question ’I get paid for sex because my friends do and they encourage me'.

¶Respondent considers himself/herself to be in more than one ongoing sexual relationship at the date of interview.

^a^Among men aged 17–54 years and women aged 15–44 years who were regular household members and stayed in the household for at least 4 nights in the last month before interview.

Estimates of sexual partners, condom use and commercial sex for men and women who have started sex.

The Manicaland cohort provided the first evidence for declines in HIV prevalence occurring within the general population in Zimbabwe associated with reductions in sexual risk behaviours.^13^ In other studies, data from the cohort have been used to assess the effectiveness of national HIV prevention programmes in reducing sexual risk behaviour.^33–35^

The inter-relationships between migration and HIV in Manicaland are complex and not fully understood.^36 37^ However, while high internal population mobility may have driven the early spread of HIV infection into and within rural areas of Zimbabwe2—and therefore contributed to the size of the national epidemic—the subsequent extensive out-migration from Zimbabwe was probably not a major factor in the decline in HIV prevalence that occurred from the late 1990s.^16 38^

### The demographic impact of a generalised HIV epidemic

Data from the Manicaland cohort were used to test early mathematical model predictions that HIV epidemics could eliminate the high rates of natural population increase (≥3% per annum) seen in sub-Saharan African countries in the 1980s.^39–41^ By the late 1990s, adult mortality in Manicaland was much higher among HIV-infected individuals (82/1000 person-years) than in uninfected individuals (7.2/1000 person-years) and the demographic impact was dramatic. However, even in the worst affected areas (towns with HIV prevalence of 33%), population growth remained positive, falling by two-thirds from 2.9% to 1.0%.^42^ Using the cohort data, we demonstrated substantial reductions in fertility among HIV-infected women^43^ and large increases in orphanhood^44^ (and associated risks of HIV infection and poor health in orphaned adolescents)^45–47^. Increases in coverage of ART from 2.3% in 2006–2008 to 23.4% in 2009–2011 and 38.2% in 2012–2013 reduced death rates (figure 3)^48^ but, as yet, have not prevented HIV-associated subfertility within the general population.^22^

### The role of social capital in HIV control in Zimbabwe

Several studies have used data from the Manicaland cohort and, in some cases, qualitative data from the same populations to improve understanding of the underlying socioeconomic drivers of the spread of HIV infection. These have included studies on poverty and the influence of economic crises on patterns of HIV risk,^4 49^ and on patterns and effects of HIV stigma,^50^ masculinity,^51 52^ religion,^14 53^ and female sex work.^54^ An unusual feature has been the innovative mixed-methods research done to describe local patterns of social capital. Strong evidence was found for associations between female participation in a range of different types of local community groups and reductions in HIV risk^55^ and faster uptake of new services including HIV testing and prevention of mother-to-child transmission of HIV services.^56^ The research developed the notion of HIV-competent communities^57^ and highlighted the importance of community leadership and participation as a key factor in the success of HIV control interventions.^58 59^

A full list of publications is available from the Manicaland Centre for Public Health Research website (see above).

## Strengths and limitations

The Manicaland cohort is one of the handful of long-running, large-scale, General Population HIV Sero-Surveys conducted in sub-Saharan African countries with widespread epidemics that constitute a major resource for evaluating the population-level impact of HIV control strategies.^60^ A major strength of the Manicaland cohort is its comprehensive and consistent approach to measurement of trends in HIV prevalence, HIV incidence, HIV-associated mortality and all-cause mortality, sexual risk behaviours and health-seeking behaviours, and the coverage and effects of national HIV control programmes over time. The study is also unusual in its inclusion of a parallel ANC Survey,^22^ in its use of validated methods to improve the quality of data on sexual behaviour^61^ and in its use of mathematical models in interpreting the wider implications of the findings.^31 62^

A weakness of studies that focus on localised areas can be difficulty in establishing whether findings are generalisable to the national population. In the Manicaland cohort, this has been addressed partially by including four of the main socioeconomic strata found in Zimbabwe. Triangulation of results with data from national sources shows that the overall levels and trends in the HIV epidemic observed in the study sites have been similar to those seen nationally.^16 63 64^ The broader generalisability of the data on sexual behaviour patterns and trends has been explored in collaborative work with other general population studies in sub-Saharan Africa in the ALPHA network^65 66^ and through studies using mathematical models.^67^ Specific weaknesses of the cohort that we hope to address in the future include the age limit (55 years) for participation, and the length of (18–24 months) and time intervals between (2–3 years) rounds of follow-up. The latter means that individuals who move into and out of the study areas between rounds of the survey, may be missed and that measurement of some key variables including mortality can be subject to recall and misclassification bias.

## Collaborations and future directions

The Manicaland cohort has provided a valuable resource and platform for the design and implementation of a number of trials of HIV control interventions and collaborative projects led by independent researchers. These include trials of peer education among female sex workers and their clients (1998–2003)^3^ and conditional and unconditional cash transfers to support orphans and vulnerable children (2009–2011),^68^ studies on HIV and migration,^37 69^ and innovative studies on HIV competent schools^70 71^ and on patterns of social contacts that influence the spread of infectious diseases in children.^72^

Subject to funding availability, we plan to extend the cohort to provide data on the implementation and impact of current and future HIV control programmes including primary prevention interventions and programmes to address major comorbidities associated with the ageing of HIV epidemics. We would welcome proposals for further collaborative projects related to this work.
