# Supplementary material for: Documenting and explaining the HIV decline in east Zimbabwe: the Manicaland General Population Cohort
Source: BMJ Open. 2017 Oct 6;7(10):e015898. doi: 10.1136/bmjopen-2017-015898 (PMC5639985; doi:10.1136/bmjopen-2017-015898)
Supplement: Supplementary material 1 [file bmjopen-2017-015898supp001.pdf]

**Cohort Profile: Documenting and Explaining the HIV Decline in East Zimbabwe: the Manicaland General Population Cohort**

# Supplementary Material

**Table S1** Household participation rates by place of residence in six rounds of the Manicaland cohort survey, 1998-2013

| | Census round | | | | | |
|---|---|---|---|---|---|---|
| | 1998-2000[a] | 2001-2003 | 2003-2005 | 2006-2008[b] | 2009-2011[b,c] | 2012-2013[b,c,d] |
| | N | N | N | N | N | N |
| Number listed (incl. 'NO' households[e]) | 8386 | 7189 | 9773 | 12668 | 13453 | 8931 |
| Characteristic | % | % | % | % | % | % |
| Overall response rate | 98.2 | 97.1 | 95.4 | 93.7 | 98.0 | 90.9 |
| Residence | | | | | | |
| Small towns | 99.3 | 99.3 | 96.5 | 91.2 | 98.1 | 87.8 |
| Agricultural estates | 99.4 | 93.7 | 92.2 | 91.9 | 96.7 | 88.7 |
| Subsistence farming villages[f] | 97.0 | 98.7 | 97.1 | 95.4 | 98.5 | 93.0 |
| Households selected for cohort interviews | - | - | - | 93.3 | 98.0 | 90.0 |
| Round 6 sites[d] | 98.2 | 97.4 | 95.9 | 93.8 | 98.1 | 90.9 |

[a] The estimates of participation rates at baseline are likely to be over-estimates because fears of Satanism led local community guides to avoid directing the fieldw orkers to some households

[b] Figures are show n for all households. i.e. including those w here no individual interview s w ere done

[c] Households listed but not done in the round w ere assumed to be no longer resident if no one w as at home and the household w as also not done in the previous round

[d] The number of sites enumerated in the study w as reduced in 2012-2013 (round 6) from 12 to eight

[e] 'NO' households w ere the third of households not included in the random sample of tw o-thirds of households selected for individual cohort interview s

[f] Subsistence farming villages here include the roadside settlements

**Table S2** Cohort participation rates by sex, age and place of residence among individuals (males aged 17-54 years, females aged 15-44 years[b]) in six rounds of the Manicaland cohort study, 1998-2013

| | 1998-2000[b] | 2001-2003[c] | 2003-2005[d] | 2006-2008[e] | 2009-2011[e] | 2012-2013e[e-g] |
|---|---|---|---|---|---|---|
| | N | N | N | N | N | N |
| Number listed | 11453 | 7172 | 16081 | 12086 | 14183 | 9353 |
| Number interviewed & tested | 9109 | 6269 | 13196 | 9466 | 11187 | 6826 |
| Characteristic | % | % | % | % | % | % |
| Overall response rate | 79.5 | 87.4 | 82.1 | 78.3 | 78.9 | 73.0 |
| Sex | | | | | | |
| Male | 79.6 | 85.8 | 76.7 | 73.9 | 71.1 | 66.0 |
| Female | 79.5 | 88.7 | 86.2 | 81.8 | 85.1 | 78.7 |
| Age (years) | | | | | | |
| 15-24 (17-24 for males) | 76.4 | 88.0 | 82.1 | 76.2 | 75.1 | 65.9 |
| 25-34 | 84.2 | 86.8 | 83.4 | 79.6 | 80.6 | 76.9 |
| 35-44 | 81.6 | 89.6 | 84.4 | 83.3 | 84.0 | 79.8 |
| 45-54 (males only) | 75.8 | 76.7 | 65.0 | 69.9 | 76.5 | 72.7 |
| Residence | | | | | | |
| Small towns | 85.5 | 90.1 | 79.2 | 75.8 | 76.6 | 73.5 |
| Agricultural estates | 78.6 | 84.5 | 79.5 | 78.3 | 80.7 | 76.0 |
| Subsistence farming villages[h] | 78.3 | 88.6 | 84.6 | 79.1 | 78.8 | 71.6 |
| Round 6 sites[g] | 79.6 | 87.1 | 83.5 | 79.5 | 79.0 | 73.0 |

[a] Participation based on completion of interview and providing a specimen for an HIV test; individuals w ere eligible if they w ere in the eligible age-groups for males and females, they w ere regular members of the household, and they had stayed in the household for at least 4 nights in the last month

[b] These eligible age ranges w ere used for males and females in the first tw o rounds of the cohort survey and are used here for all rounds for greater consistency of comparison; in practice, the eligible age-range for the cohort w as extended to 15-54 years for both sexes from the third round of the survey

[c] At baseline, eligibility w as further limited to individuals w ho had been staying in the household at the same time one year earlier; in the first tw o rounds, one member of each marital group only w as selected at random as eligible for participation in the study

[d] In round tw o, individuals w ho had migrated into a household in the study areas since baseline w ere only treated as eligible from site 5 (out of 12 sites)

[e] From round 3 the restriction of selecting a maximum of one member per marital grouping for interview w as dropped

[f] From round 4, participation w as restricted to eligible individuals in a random sample of tw o-thirds of households

[g] In round 6, the number of study sites w as reduced from 12 to eight (tw o agricultural estates and tw o subsistence farming areas w ere dropped)

[h] Subsistence farming villages here include the roadside settlements

**Table S3** Follow-up rates by sex, age and place of residence among individuals (males aged 17-54 years, females aged 15-44 years) in six rounds of the Manicaland cohort study, 1998-2013

| | Follow-up rates[a] (deaths & out-migrants treated as lost-to-follow-up) | | | | | Follow-up rates (excluding deaths & out-migrants between rounds) | | | | |
|---|---|---|---|---|---|---|---|---|---|---|
| | 2001-2003[c] | 2003-2005 | 2006-2008[d] | 2009-2011[d] | 2012-2013[d,e] | 2001-2003 | 2003-2005 | 2006-2008 | 2009-2011 | 2012-2013 |
| | N | N | N | N | N | N | N | N | N | N |
| Number listed | 9109 | 6269 | 8231 | 9466 | 7824 | 5461 | 3942 | 5080 | 5152 | 5434 |
| Number interviewed & tested | 4817 | 3802 | 4005 | 4448 | 4183 | 4817 | 3802 | 4005 | 4448 | 4183 |
| Characteristic[b] | % | % | % | % | % | % | % | % | % | % |
| Overall response rate | 52.9 | 60.6 | 48.7 | 47.0 | 53.5 | 88.2 | 96.4 | 78.8 | 86.3 | 77.0 |
| Sex | | | | | | | | | | |
| Male | 47.3 | 56.2 | 44.5 | 42.9 | 48.3 | 86.8 | 96.2 | 76.0 | 80.9 | 71.0 |
| Female | 57.6 | 64.1 | 51.4 | 49.9 | 56.8 | 89.2 | 96.6 | 80.5 | 90.0 | 80.8 |
| Age (years) | | | | | | | | | | |
| 15-24 (17-24 for males) | 40.2 | 47.6 | 33.5 | 30.3 | 36.1 | 83.4 | 95.4 | 66.4 | 77.6 | 63.4 |
| 25-34 | 57.9 | 65.9 | 57.0 | 51.9 | 59.2 | 89.4 | 96.6 | 84.5 | 88.2 | 81.6 |
| 35-44 | 73.4 | 76.4 | 67.8 | 69.9 | 71.3 | 93.1 | 97.4 | 88.9 | 92.7 | 85.8 |
| 45-54 (males only) | 64.3 | 76.1 | 62.1 | 70.0 | 67.9 | 91.4 | 97.2 | 85.4 | 90.8 | 81.7 |
| Residence | | | | | | | | | | |
| Small towns | 43.9 | 50.4 | 41.5 | 42.9 | 50.4 | 87.1 | 92.1 | 82.4 | 83.4 | 76.3 |
| Agricultural estates | 54.3 | 63.9 | 49.3 | 47.4 | 55.5 | 85.9 | 97.4 | 76.4 | 86.6 | 79.2 |
| Subsistence farming villages | 55.0 | 61.7 | 50.3 | 48.0 | 54.2 | 90.1 | 97.0 | 79.4 | 87.0 | 76.4 |
| HIV infection status | | | | | | | | | | |
| HIV+ | 52.3 | 60.2 | 46.3 | 48.5 | 61.9 | 89.1 | 95.5 | 82.7 | 88.2 | 82.4 |
| HIV- | 53.1 | 60.8 | 49.2 | 46.7 | 51.8 | 87.9 | 96.7 | 78.0 | 85.9 | 75.8 |
| Round 6 sites[e] | 52.9 | 60.8 | 49.3 | 45.6 | 53.5 | 88.7 | 96.4 | 78.6 | 85.9 | 77.0 |

[a] Follow-up rates based on individuals who were eligible in the previous round using the same criteria as for the participation rates (i.e. excluding visitors, non-regular members, and those who stayed in the household for less than 4 nights in the last month); including those who no longer met the eligibility criteria for initial enrolment (i.e. by age and household residence)

[b] Status at previous round

[c] 177 participants at baseline who had migrated out of the study areas by round two and were followed up and participated are treated here as lost-to-follow-up at round 2

[d] From round 4, participation was restricted to eligible individuals in a random sample of two-thirds of households

[e] In round 6, the number of study sites was reduced from 12 to eight

**Table S4** Information collected in serial cros-sectional antenatal HIV surveillance conducted in parallel with the Manicaland Cohort survey

| Survey instrument | Survey rounds† | Scope of question | Specific information |
|---|---|---|---|
| **Antenatal clinic survey** | | | |
| Service availability | Round 4→ | Pregnant women attending | HIV testing, PMTCT staff, regimens and drug supplies |
| Background characteristics | All | antenatal clinics in study areas 100-120 per study site in each | Age, education, residence, age at first sex, marital status, parity, general health, STDs |
| HIV testing | Round 4→ | round | HIV testing (with/without partner), HIV test result |
| Care and ARV treatment | Round 4→ | HIV-positive women | WHO stage assessment, ART uptake, PMTCT uptake / adherence |
| HIV infection status | All | All | Combaids HIV-1 / HIV-2 dipstick test |

STDs, sexually transmitted diseases; ART, antiretroviral therapy; PMTCT, prevention of mother-to-child transmission of HIV infection

† Dates of the ANC surveys: Round 1: July 1998 to January 2000; round 2: August 2001 to July 2003; round 3: August 2003 to August 2005; round 4: August 2006 to November 2008; round 5: October 2009 to July 2011; round 6: July 2012 to November 2013

‡ Eligibility for the ANC surveys: pregnant women attending for a routine ANC check-up (not necessarility the first) at a health facility in the study areas for a pregnancy during the periods of the survey visits to each site in the Manicaland Cohort. The study sites in each round were the same as for the Manicaland Cohort (see main text)
